# Resistance to Cleavage of Core–Shell Rubber/Epoxy Composite Foam Adhesive under Impact Wedge–Peel Condition for Automobile Structural Adhesive

**DOI:** 10.3390/polym11010152

**Published:** 2019-01-17

**Authors:** Jong-Ho Back, Dooyoung Baek, Jae-Ho Shin, Seong-Wook Jang, Hyun-Joong Kim, Jong-Hak Kim, Hong-Kyu Song, Jong-Won Hwang, Min-Jae Yoo

**Affiliations:** 1 Laboratory of Adhesion and Bio-Composites, Program in Environmental Materials Science, College of Agriculture and Life Science, Seoul National University, Seoul 08826, Korea; beak1231@snu.ac.kr (J.-H.B.); baek.s.dy@snu.ac.kr (D.B.); pass2462@snu.ac.kr (J.-H.S.); jangsw0202@snu.ac.kr (S.-W.J.); 2 Research Institute of Agriculture and Life Sciences, College of Agriculture and Life Sciences, Seoul National University, Seoul 08826, Korea; 3 Unitech Co., Ltd., Byeolmang-ro 459beon-gil 45, Gyeonggi-do 15598, Korea; jh.kim@unitech99.co.kr (J.-H.K.); hongq@unitech99.co.kr (H.-K.S.); 4 Kukdo Chemical Co., Ltd., Gasandigital 2-ro, Seoul 08588, Korea; pac3@kukdo.com; 5 Korea Research Institute of Chemical Technology, 141 Gajeongro, Daejeon 34114, Korea; yoomin@krict.re.kr

**Keywords:** epoxy composite foam adhesive, core–shell rubber, impact wedge–peel test, automobile structural adhesives

## Abstract

Epoxy foam adhesives are widely used for weight reduction, watertight property, and mechanical reinforcement effects. However, epoxy foam adhesives have poor impact resistance at higher expansion ratios. Hence, we prepared an epoxy composite foam adhesive with core–shell rubber (CSR) particles to improve the impact resistance and applied it to automotive structural adhesives. The curing behavior and pore structure were characterized by differential scanning calorimetry (DSC) and X-ray computed tomography (CT), respectively, and impact wedge–peel tests were conducted to quantitatively evaluate the resistance to cleavage of the CSR/epoxy composite foam adhesives under impact. At 5 and 10 phr CSR contents, the pore size and expansion ratio increased sufficiently due to the decrease in curing rate. However, at 20 phr CSR content, the pore size decreased, which might be due to the steric hindrance effect of the CSR particles. Notably, at 0 and 0.1 phr foaming agent contents, the resistance to cleavage of the adhesives under the impact wedge–peel condition significantly improved with increasing CSR content. Thus, the CSR/epoxy composite foam adhesive containing 0.1 phr foaming agent and 20 phr CSR particles showed high impact resistance (*E*_C_ = 34,000 mJ/cm^2^) and sufficient expansion ratio (~148%).

## 1. Introduction

An epoxy foam adhesive is an epoxy containing a foaming agent that generates a gas inside the epoxy resin or expands upon heat treatment. After heat treatment, the epoxy foam adhesive is cured and foamed, simultaneously filling the gap between two substrates and binding them [1,2]. Through this process, the epoxy foam adhesive provides weight reduction, watertight property, and reinforcement effects, and can thus be applied to structural adhesives in automobiles [1].

As the mechanical strength of an epoxy foam adhesive weakens through an increase in the expansion ratio [3], it is necessary to improve the mechanical strength while preserving the expansion ratio. Particularly, since the impact resistance of an epoxy foam adhesive is important for its application to structural adhesives in automobiles, epoxy composite foam adhesives containing an additive that improves impact resistance are required [4].

Rubber particles have been widely used to enhance the impact resistance of epoxy composites [5,6,7,8]. However, the poor dispersion and aggregation of rubber particles in a composite decreases the impact resistance at a high content of rubber particles. Therefore, core–shell rubber (CSR) particles, in which rubber particles form the core structure and a polymer forms the shell, have been developed [9]. Using CSR particles in a composite rather than rubber particles, the dispersion of CSR particles in the composite can be improved and impact resistance can be enhanced [9,10,11].

The pore structure of epoxy composite foams is typically characterized by two-dimensional analysis, such as scanning electron microscopy (SEM) [3,12,13,14,15]. Two-dimensional analysis can be used to observe only the sample surface, and the investigation of the internal pore structure necessitates a destructive evaluation of the epoxy composite foam adhesive. However, X-ray computed tomography (CT) can nondestructively characterize the internal pore structure of polymeric foams [1,16,17] and can quantitatively evaluate the average pore size, standard deviation of pore size, porosity, and expansion ratio.

Further, the impact resistance of an epoxy composite containing CSR particles has been conventionally evaluated by a ballistic impact test [10], izod impact test [11], etc. By contrast, to evaluate the impact resistance of structural adhesives, a test specimen is adhesively bonded and impact is applied to the specimen using instruments such as an Izod impact tester [4,11,18], impact wedge–peel tester [19,20], and servohydraulic tester [21].

In this study, epoxy composite foam adhesives containing epoxy resin, a foaming agent, and CSR particles were prepared, and their pore structure was characterized by X-ray CT. We used an impact wedge–peel tester and their resistance to cleavage of the epoxy composite foam adhesive under impact condition. We investigated the effect of CSR particles on the pore structure and impact resistance of the epoxy composite foam adhesive containing different amounts of foaming agent and suggested an optimal content of CSR particles to achieve a high expansion ratio and impact resistance.

## 2. Materials and Methods

### 2.1. Materials

**Figure 1:**
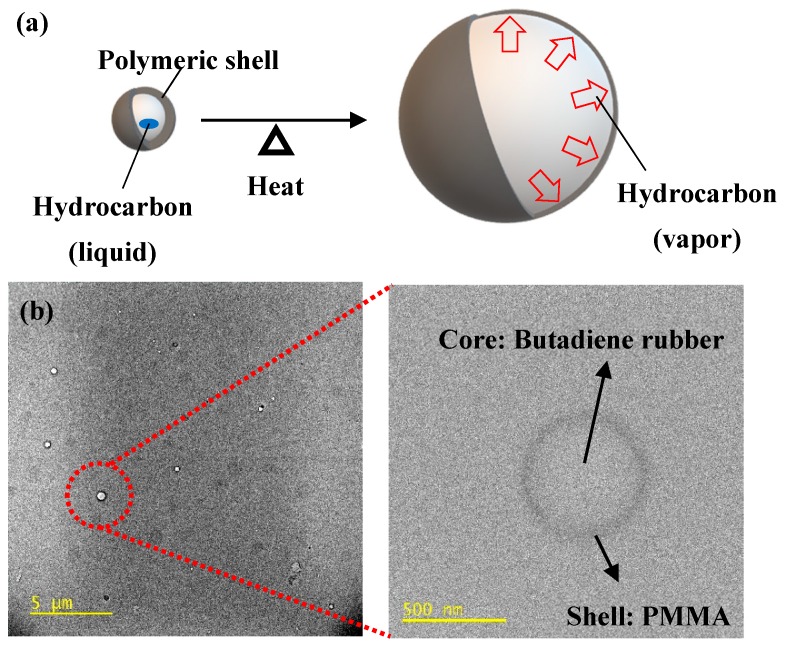
(**a**) Foaming mechanism of expandable microcell foaming agent and (**b**) TEM images of core–shell rubber (CSR) particle.

Two epoxy resins were used to prepare epoxy composite foams: Bisphenol-A diglycidyl epoxy resin (YD-128, Kukdo Chemical Co., Ltd., Seoul, Korea) and urethane-modified epoxy resin (UME, Kukdo Chemical Co., Ltd., Seoul, Korea). A dicyandiamide curing agent (Dyhard 100S, AlzChem, Trostberg, Germany) and a latent accelerator (Dyhard UR500, AlzChem, Trostberg, Germany) were blended with the epoxy resins for curing. The CSR particles were composed of butadiene rubber cores and poly(methyl methacrylate) shells. We used CSR particles (35 wt %) predispersed in bisphenol-A diglycidyl epoxy resin (KDAD-1760, Kukdo Chemical Co., Ltd., Seoul, Korea). Calcium carbonate (CaCO_3_, 10CN, OMYA, Oftringen, Switzerland) and expandable microcell (F-360, Matsumoto Yushi-Seiyaku Co., Ltd., Osaka, Japan) were used as filler and a foaming agent, respectively. Detailed information of the materials employed in this work is presented in Table 1. The foaming mechanism of the expandable microcell and the TEM images of the CSR particles are shown in Figure 1.

### 2.2. Curing and Foaming of CSR/Epoxy Composite Foam Adhesive

Materials were blended, maintaining the ratio of total equivalent weight of epoxy to curing agents as 1.00 (Table 2). Different amounts of the CSR mixture were blended so that the CSR content in the composites varied as 0, 5, 10, and 20 phr. Further, CaCO_3_ was added so that the total weight of CSR particles and CaCO_3_ was 20 phr. Moreover, the foaming agent content in the samples was varied as 0, 0.1, and 1 phr. All the samples were cured and foamed at 170 °C for 40 min.

### 2.3. Differential Scanning Calorimetry (DSC)

DSC (DSC Q200, TA Instruments-Waters Korea Ltd., Seoul, Korea) was performed to compare the curing behaviors of the epoxy composite foams. The heat flow of exothermic curing reaction was measured during the DSC run in the temperature range of 60–240 °C at a constant heating rate of 5 °C/min.

### 2.4. X-ray Computed Tomography

The pore structure was characterized by X-ray CT (Skyscan 1272, Bruker Korea Co., Ltd., Gyeonggi-do, Belgium). The X-ray head (50 kV) was rotated around the epoxy composite foam and tomographic images were captured every 0.6°. These tomographic images were collected and converted into 3D images. The average pore size, standard deviation of pore size, and porosity were evaluated by the software (CT Analyzer, Bruker Korea CO., Ltd., Gyeonggi-do, Belgium), and the expansion ratio was calculated by Equation (1).

(1) $$\mathrm{Expansion}\;\mathrm{ratio}\;\left(\%\right)=\frac{V_{\mathrm{total}}}{V_{\mathrm{total}}-V_{\mathrm{pore}}}\;\times \;100=\;\frac{100}{100-porosity\;\left(\%\right)}\;\times \;100,$$

where *V*_pore_ and *V*_total_ represent the total volume of pores and the measured region, respectively. 

### 2.5. Impact Wedge–Peel Test

**Figure 2:**
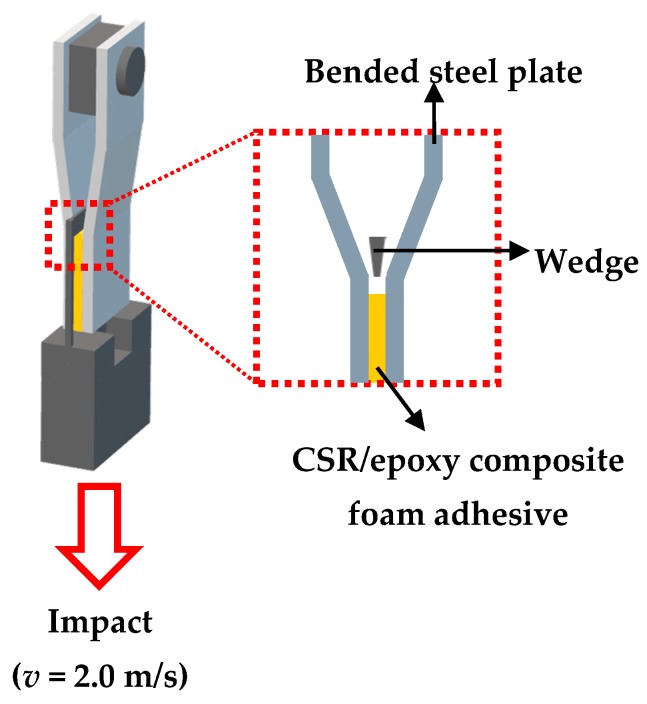
Schematic illustration of specimen prepared for impact wedge–peel test.

An impact wedge–peel test was performed according to ISO 11343 standard to compare the resistance to cleavage of the CSR/epoxy composite foam adhesives under impact using a drop weight tester (Dyntaup® Model 9250HV, Instron, Norwood, MA, USA). Specimens for the impact wedge–peel test were prepared as shown in Figure 2. Two bent steel plates (length: 90 mm, width: 20 mm, thickness: 1.6 mm, material: CR340) were bonded using the CSR/epoxy foam composite adhesive (area: 20 × 20 mm^2^, thickness: 0.2 mm), and the force was measured when the adhesive layer was cleaved by the wedge at a velocity (*v*) of 2.0 m/s.

**Figure 3:**
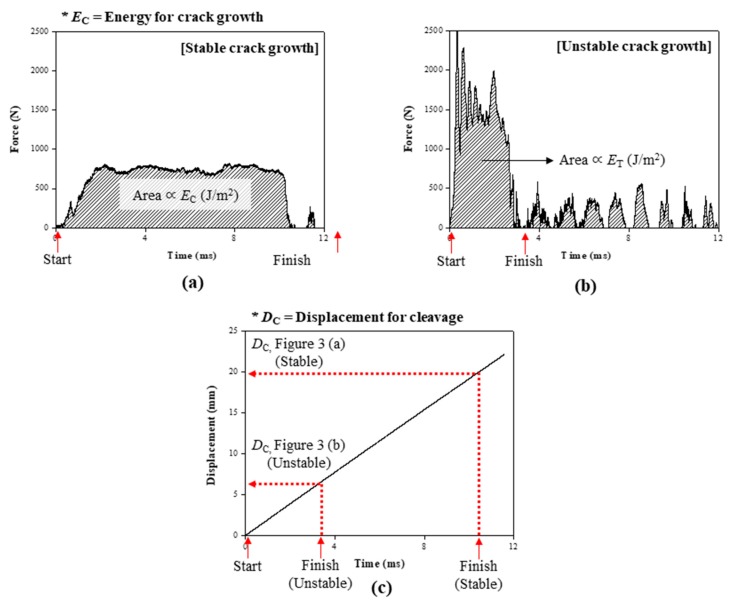
Results of impact wedge–peel test: Time–force curves for (**a**) stable crack growth and (**b**) unstable crack growth. (**c**) Time–displacement curve.

As shown in Figure 3, a force–time curve can be obtained by the impact wedge–peel test. According to the shape of the curve, crack growth can be classified into two types: Stable and unstable crack growth. While stable crack growth has a constant region of cleavage force, for unstable crack growth, cleavage occurred in an instant without a constant region of force (Figure 3a,b, respectively). The area of the force–time curve is proportional to the energy of crack growth (*E*_C_), which quantitatively represents the impact resistance. Displacement for cleavage (*D*_C_) is determined by the displacement at the finish point of the cleavage.

## 3. Results and Discussion

### 3.1. Curing Behavior of CSR/Epoxy Composite

**Figure 4:**
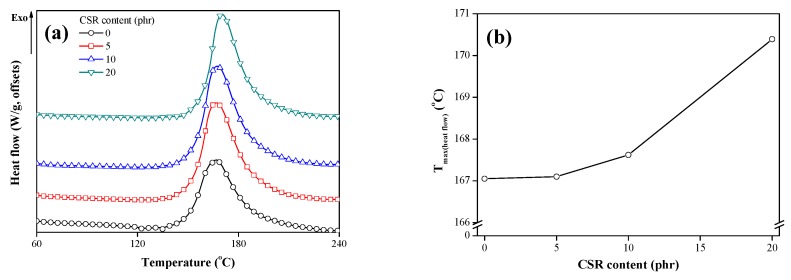
(**a**) Differential scanning calorimetry (DSC) curves of CSR/epoxy composites and (**b**) maximum temperature of heat flow.

The curing behavior of the CSR/epoxy composite was studied by DSC (Figure 4). As the curing of epoxy is an exothermic reaction, all the samples exhibited an exothermic peak, and the maximum temperature of heat flow (*T*_max (heat flow)_) was plotted as a function of CSR content. Notably, as the CSR content increased, *T*_max (heat flow)_ got higher. This indicates that the addition of CSR particles retarded the curing reaction of epoxy due to the steric hindrance effect of the CSR particles in the CSR/epoxy composite.

### 3.2. Pore Structure of CSR/Epoxy Composite Foam Adhesive

**Figure 5:**
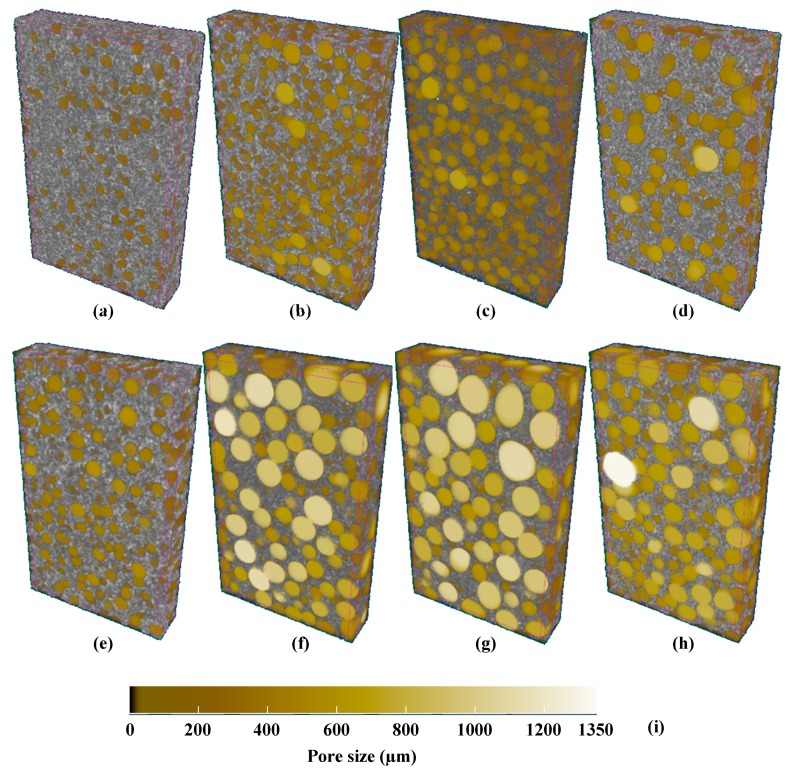
3D images of CSR/epoxy composite foam adhesives: (**a**) CSR 0/FA 0.1, (**b**) CSR 5/FA 0.1, (**c**) CSR 10/FA 0.1, (**d**) CSR 20/FA 0.1, (**e**) CSR 0/FA 1, (**f**) CSR 5/FA 1, (**g**) CSR 10/FA 1, and (**h**) CSR 20/FA 1. (**i**) Color scale indicator for pore size (FA = Foaming agent).

**Figure 6:**
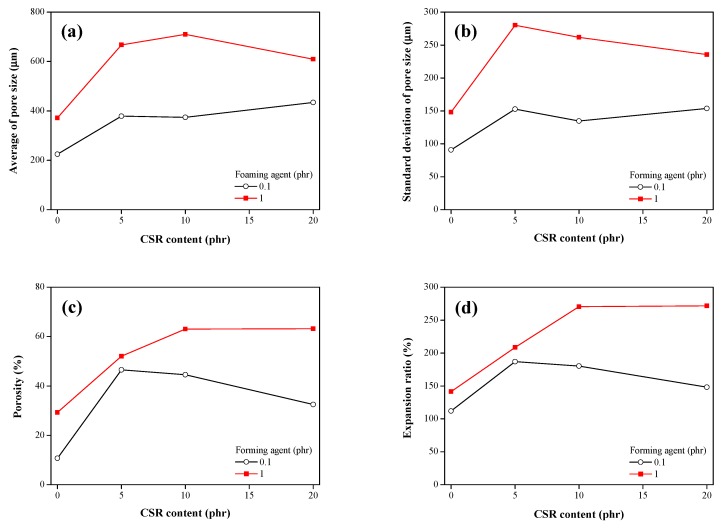
(**a**) Average and (**b**) standard deviation of pore size, (**c**) porosity, and (**d**) expansion ratio of CSR/epoxy composite foam adhesives.

The pore structures of the CSR/epoxy composite foam adhesives were analyzed by X-ray CT. As shown in Figure 5, the pore structure could be investigated from the 3D images of the CSR/epoxy composite foam adhesives, where the pore sizes are assigned a color gradation. Notably, the pore size changed with the CSR content, which indicated that the addition of CSR particles affected the expansion of the foaming agent. To quantitatively compare the pore structures of the CSR/epoxy composite foam adhesives, many parameters, including the average pore size, standard deviation of pore size, porosity, and expansion ratio, were evaluated (Figure 6). The pore size and expansion ratio for 1 phr foaming agent is higher than those for 0.1 phr foaming agent. Compared to 0 phr CSR content, at 5 and 10 phr CSR contents, the pore size and expansion ratio increased sufficiently due to the decrease in curing rate. It has been reported that the curing behavior affects the pore growth and that the expansion ratio increases at a slow curing speed [1,14]. However, although curing was retarded at 20 phr CSR content, the pore size and expansion ratio decreased. This might have resulted from the steric hindrance effect of the CSR particles, which spatially prevented the expansion of the foaming agent [1].

### 3.3. Resistance to Cleavage of Adhesive under Impact Wedge–Peel Condition

**Figure 7:**
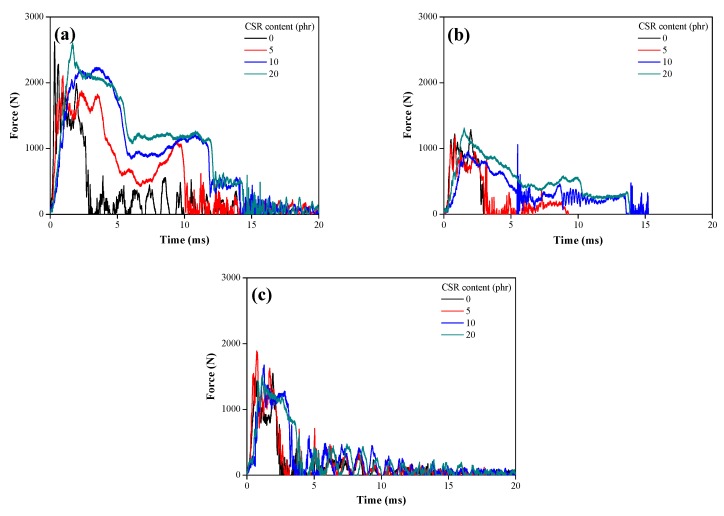
Time–force curves of impact wedge–peel test: Foaming agent content of (**a**) 0 phr, (**b**) 0.1 phr, and (**c**) 1 phr.

Under the impact wedge–peel condition, the impact force was measured for 20 ms (Figure 7). With an increase in CSR content, the force was increased and sustained for a longer period, indicating that the CSR particles significantly improved the impact resistance of the epoxy composite foam adhesives. On the other hand, as the foaming agent content increased, the cleavage time decreased drastically, suggesting that the CSR/epoxy composite foam adhesive became fragile.

As shown in Table 3, the type of crack growth and the displacement for cleavage were investigated to compare the impact resistance. With an increase in the foaming agent content, the CSR/epoxy composite foam adhesives exhibited unstable crack growth and a short displacement for cleavage (*D*_C_). It indicates that the impact resistance deteriorated with increasing foaming agent content. As the CSR content increased, the impact resistance of the CSR/epoxy composite foam adhesive improved dramatically, resulting in an increase in *D*_C_ and changing the type of crack growth from unstable to stable crack growth.

**Figure 8:**
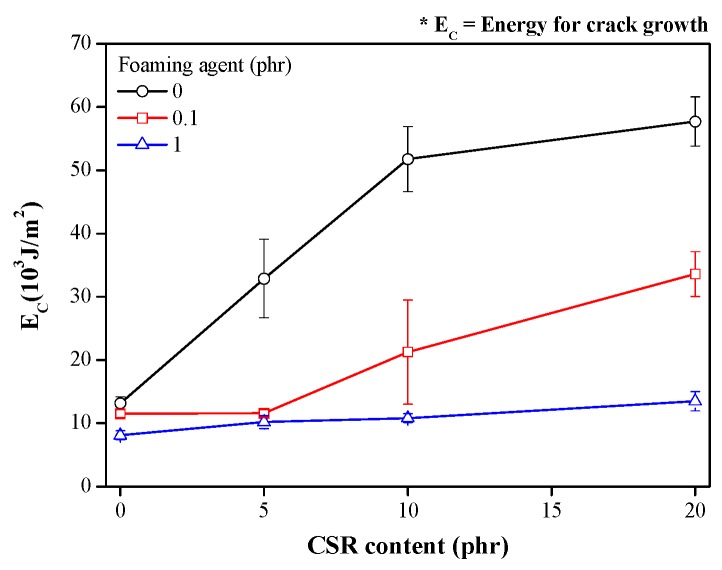
Energy for crack growth of CSR/epoxy composite foam adhesives.

Additionally, by comparing the energy for crack growth (*E*_C_), we quantitatively evaluated the resistance to cleavage of the CSR/epoxy composite foam adhesives under the impact wedge–peel condition. As shown in Figure 8, at foaming agent contents of 0 and 0.1 phr, the *E*_C_ was significantly enhanced by the addition of CSR particles, indicating an improvement in impact resistance. However, as the foaming agent content increased to 1 phr, the *E*_C_ hardly increased, which suggests that the impact resistance effectively improved at low foaming agent contents (0 and 0.1 phr).

Notably, for the CSR/epoxy composite foam adhesive containing 0.1 phr foaming agent and 20 phr CSR particles, the *E*_C_ (34,000 J/m^2^) was more than two times that of the adhesive containing no CSR particle (12,000 J/m^2^). In addition, the type of crack growth changed from unstable to stable crack propagation by the addition of 20 phr CSR particles into the sample containing 0.1 phr foaming agent. Moreover, the expansion ratio of the adhesive containing 0.1 phr foaming agent and 20 phr CSR particles increased, compared to the adhesive containing no CSR particle; this indicated that a simultaneous increase in both the expansion ratio (~148%) and impact resistance was achieved.

## 4. Conclusions

CSR/epoxy composite foam adhesives were prepared with different amounts of foaming agent and CSR particles. With increasing CSR content, the curing reaction retarded, which affected the growth of the pores. The pore structure, pore size, porosity, and expansion ratio were determined by X-ray CT. The expansion ratio for 1 phr foaming agent was higher than that for 0.1 phr foaming agent. At 5 and 10 phr CSR content, the pore size and expansion ratio increased by decrease in curing rate, but, at 20 phr CSR content, the pore size and expansion ratio decreased due to the steric hindrance effect of the CSR particles. The impact resistance of the CSR/epoxy composite foam adhesive was compared in terms of *E*_C_. It was significantly enhanced by the addition of CSR particles at 0 and 0.1 phr foaming agent. However, at 1 phr foaming agent, the *E*_C_ was hardly improved by the addition of CSR particle, indicating that the improvement in impact resistance is effective only at low foaming agent contents (0 and 0.1 phr). For the CSR/epoxy composite foam adhesives containing 0.1 phr foaming agent and 20 phr CSR particles, a simultaneous increase in both the expansion ratio (~148%) and impact resistance (*E*_C_ = 34000 mJ/cm^2^) was achieved. A limitation of this study is that we only focused on the impact resistance of the CSR–epoxy composite foam adhesives at room temperature. Since CSR particles can improve impact resistance at low temperatures, it is necessary to investigate the impact resistance of CSR/epoxy composite foam adhesives at low temperatures in future research.

## Figures and Tables

**Table 1:** Materials used for preparing epoxy composite foam adhesive.

Materials	Composition	Abbreviation	Equivalent Weight (g/eq)
Epoxy	Bisphenol-A diglycidyl epoxy	BPA-E	187
	Urethane-modified epoxy	UME	475
Curing agent	Dicyandiamide	CA-1	21
	Substituted urea	CA-2	3
CSR + Epoxy	CSR in epoxy resin (35 wt %)	CSR mixture	287.7

**Table 2:** Composition of epoxy composites.

Sample	BPA-E (g)	CSR Mixture (g)	CaCO_3_ (g)	Foaming Agent (g)
CSR 0/FA 0	14.96	0	6.03	0
CSR 5/FA 0	12.16	4.31	4.52	0
CSR 10/FA 0	9.36	8.62	3.02	0
CSR 20/FA 0	3.76	17.23	0	0
CSR 0/FA 0.1	14.96	0	6.03	0.032
CSR 5/FA 0.1	12.16	4.31	4.52	0.032
CSR 10/FA 0.1	9.36	8.62	3.02	0.032
CSR 20/FA 0.1	3.76	17.23	0	0.032
CSR 0/FA 1	14.96	0	6.03	0.32
CSR 5/FA 1	12.16	4.31	4.52	0.32
CSR 10/FA 1	9.36	8.62	3.02	0.32
CSR 20/FA 1	3.76	17.23	0	0.32

* Contents of urethane-modified epoxy resin (UME), CA-1, and CA-2 were set as 15.20, 1.51, and 0.12 g, respectively.

**Table 3:** Type of crack growth and displacement for cleavage of CSR/epoxy composite foam adhesives.

Foaming Agent Content (phr)	CSR Content (phr)	Type of Crack Growth	*D_C_ (mm)
0	0	Unstable	9.94 (±7.16)
	5	Unstable/Stable	22.11 (±5.17)
	10	Stable	26.30 (±1.15)
	20	Stable	26.57 (±0.93)
0.1	0	Unstable	9.23 (±5.53)
	5	Unstable	10.88 (±6.34)
	10	Unstable/Stable	22.40 (±7.81)
	20	Stable	28.87 (±0.55)
1	0	Unstable	5.85 (±1.22)
	5	Unstable	7.29 (±2.23)
	10	Unstable	8.45 (±1.63)
	20	Unstable	13.27 (±4.46)

* *D*_C_ = Displacement for cleavage.
